# Multi-scale information fusion network with label smoothing strategy for corneal ulcer classification in slit lamp images

**DOI:** 10.3389/fnins.2022.993234

**Published:** 2022-11-24

**Authors:** Linquan Lv, Mengle Peng, Xuefeng Wang, Yuanjun Wu

**Affiliations:** ^1^Anhui Finance and Trade Vocational College, Hefei, Anhui, China; ^2^Department of Mechanical and Energy Engineering, Zhejiang University of Science and Technology, Hangzhou, Zhejiang, China

**Keywords:** corneal ulcer classification, multi-scale information fusion, label smoothing, deep learning, fluorescein staining slit lamp images

## Abstract

Corneal ulcer is the most common symptom of corneal disease, which is one of the main causes of corneal blindness. The accurate classification of corneal ulcer has important clinical importance for the diagnosis and treatment of the disease. To achieve this, we propose a deep learning method based on multi-scale information fusion and label smoothing strategy. Firstly, the proposed method utilizes the densely connected network (DenseNet121) as backbone for feature extraction. Secondly, to fully integrate the shallow local information and the deep global information and improve the classification accuracy, we develop a multi-scale information fusion network (MIF-Net), which uses multi-scale information for joint learning. Finally, to reduce the influence of the inter-class similarity and intra-class diversity on the feature representation, the learning strategy of label smoothing is introduced. Compared with other state-of-the-art classification networks, the proposed MIF-Net with label smoothing achieves high classification performance, which reaches 87.07 and 83.84% for weighted-average recall (W_R) on the general ulcer pattern and specific ulcer pattern, respectively. The proposed method holds promise for corneal ulcer classification in fluorescein staining slit lamp images, which can assist ophthalmologists in the objective and accurate diagnosis of corneal ulcer.

## Introduction

Corneal ulcer is a serious blinding eye disease, which is one of the main causes of corneal blindness (Smith, 2004; Deswal et al., 2017; Lopes et al., 2019). In addition, it is an inflammatory or more serious infectious corneal disease involving disruption of the stroma epithelial layer, which can cause great pain to the patient and may cause severe vision loss or even blindness (Cohen et al., 1987; Diamond et al., 1999; Manikandan et al., 2019).

Standardized screening, timely diagnosis and early treatment are effective ways to reduce the blindness rate of corneal ulcer. Fluorescent staining is often used to observe the integrity of the ocular surface, especially the integrity of the cornea. Therefore, fluorescent staining technology has become a common tool to assist ophthalmologists in diagnosing corneal ulcer, and can provide great convenience for the diagnosis and treatment of corneal ulcer (Kaufman, 1960; Schweitzer, 1967; Morgan and Carole, 2009; Kumar and Thirumalesh, 2013; Zhang et al., 2018). The current corneal ulcer prevention and treatment model is based on the ulcer’s general pattern and type grade (TG) standards, using slit lamp microscopy combined with fluorescent staining technology to examine the ocular surface of high-risk groups (Wolffsohn and Purslow, 2003; Khanal et al., 2008; Peterson and Wolffsohn, 2009). For the ulcer’s general pattern, the diagnostic procedure for corneal ulcer is based on the shape and distribution characteristics of the corneal ulcer, which can be classified into three categories, respectively, corresponding to point-like corneal ulcers, point-flaky mixed corneal ulcers and flaky corneal ulcers (Smith, 2004; Deswal et al., 2017). While the TG grading method has two components: (1) according to the specific pattern of corneal ulcers, it is divided into five categories (type0–type4), which is usually the first step for ophthalmologists to diagnose underlying disease. (2) according to the location of corneal ulcer in the cornea, it is divided into five categories (grade0–grade4). The use of fluorescent stained images to identify corneal ulcers plays an important role in formulating treatment plans. However, due to differences in subjective experience and professional knowledge, ophthalmologists have certain differences in the recognition of corneal ulcers based on fluorescent stained images. Therefore, it is very important to study an objective and accurate automatic classification method of corneal ulcers, which can assist ophthalmologists in formulating individualized drug or surgical intervention strategies.

In the past, many related works on automated or semi-automated methods for corneal ulcers are mainly for the segmentation of corneal ulcers, which is considered to be a pixel-by-pixel classification. For example, Wolffsohn and Peterson et al. applied the color extraction algorithm and edge detection algorithm of the RGB system to automatically segment the ulcer area (Wolffsohn and Purslow, 2003; Peterson and Wolffsohn, 2009), while Pritchard et al. (2003) used threshold technology to indirectly detect conjunctival hyperemia and punctate corneal ulcers. Chun et al. (2014) used RGB and hue-saturation-value (HSV) techniques to evaluate corneal staining on 100 images. Deng et al. (2018b) successively used k-means clustering, morphological operations, and region growth to achieve automatic ulcer segmentation, and they also proposed a simple linear iterative clustering (SLIC) based super-pixel method (Deng et al., 2018a). In addition, Liu et al. used the combined method of Otsu and Gaussian mixture modeling (GMM) to segment the intracorneal ulcer area on 150 images. In recent years, deep learning has received widespread global attention, and its automated analysis of ophthalmic images has also made a huge breakthrough (Liu et al., 2018; Peng et al., 2020, 2021, 2022a,b,c; Wang et al., 2020). Recently, some progress has been made in the detection and segmentation of corneal ulcers based on deep learning. For example, Sun et al. (2017) proposed a patch-based deep convolutional neural network to segment corneal ulcers. A recent study proposed a system for automatically detecting corneal ulcer disease (Akram and Debnath, 2019), which first uses the Haar cascade classifier to detect and segment the eye part of the face, and then, the convolutional neural network (CNN) is used to detect the presence of corneal ulcer disease. If there is corneal ulcer, active contour technology is used to locate and segment the ulcer area.

**FIGURE 1:**
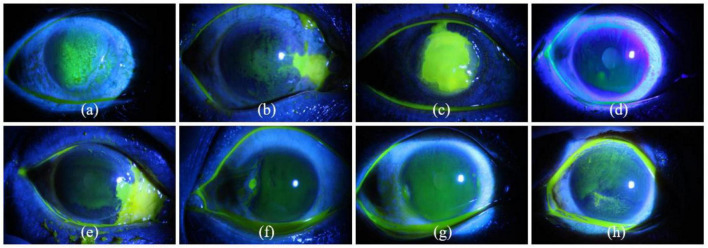
Eight examples of slit lamp images of corneal ulcers. Panels **(a–c)** represent point-like corneal ulcer, point-flaky mixed corneal ulcer, and flaky corneal ulcer according to general ulcer pattern, while panels **(d–h)** are type0, type1, type2, type3, and type4 corneal ulcers according to specific ulcer pattern.

The above studies mainly focus on the segmentation of corneal ulcer. Few studies involve the classification of corneal ulcer, which is an important reference for ophthalmologists to formulate treatment strategies. Therefore, the current paper builds upon the previous successful models and proposes a simple and effective methodology, which combines multi-scale information fusion and label smoothing strategy to achieve two classification patterns of corneal ulcer. The first is to realize the classification of corneal ulcer according to the ulcer’s general pattern, while the second is to achieve the type grading of corneal ulcers according to the specific ulcer pattern. The relevant images of corneal ulcer are shown in the Figure 1, and a detailed description of the relevant symptoms and the number of fluorescein staining images in each category is given in Table 1. It can be seen from Figure 1 and Table 1 that it is challenging to achieve accurate corneal ulcer classification mainly due to the following two reasons: (1) Corneal ulcer has complex pathological features and noise interference; (2) Different types of corneal ulcers are similar in pathological shape and distribution. In this study, for point-like corneal ulcers, point-flaky mixed corneal ulcers and type1 corneal ulcers with micro punctate, continuous down sampling of the pool layer in CNN may lead to the loss of lower resolution features related to category, which may lead to the decline of classification performance. Previous studies have shown that some shallow features can improve classification performance (Lee et al., 2015; Wang et al., 2015; Ma et al., 2019), which has attracted our attention. In addition, considering that the misclassification of corneal ulcers may be caused by images of similar but different categories in the dataset (Wan et al., 2021), we introduce label smoothing in the cross-entropy loss, which can reduce the inter-class similarity and intra-class differences (Müller et al., 2019). In short, we develop a multi-scale information fusion network with label smoothing strategy to achieve the classification of corneal ulcer. The main contributions of this paper can be summarized as follows:

**TABLE 1:** Symptoms and number of different types of corneal ulcers.

Ulcer pattern	Categories	Symptoms	Num	Total
General ulcer pattern	Point-like corneal ulcers	A large number of small ulcers are gathered and can be distributed anywhere on the cornea.	358	712
	Point-flaky mixed corneal ulcers	Between punctate and lamellar corneal ulcers, there are both punctate and lamellar ulcers in the cornea with irregular distribution.	263	
	Flaky corneal ulcers	The ulcer area is usually bright green with clear borders.	91	
Specific ulcer pattern	Type0	No ulcer of the corneal epithelium.	36	712
	Type1	Micro punctate.	78	
	Type2	Macro punctate.	40	
	Type3	Coalescent macro punctate.	10	
	Type4	Patch (>=1 mm).	548	

“Num” and “Total” represent the number of fluorescein staining images in each category and total number of fluorescein stained images.

(1) To make full use of shallow edge information and deep semantic information, a multi-scale information fuser is designed to enhance feature expression capabilities, which can improve the robustness of prediction results.
(2) Label smoothing strategy is introduced to reduce the impact of the inter-class similarity and intra-class differences between corneal ulcer images on feature representation and guide the model to learn salient features with category differences.
(3) Extensive experiments are conducted to evaluate the effectiveness of the proposed MIF-Net and the results show that the proposed MIF-Net outperforms other state-of-the-art classification networks.

The remainder of this paper is organized as follows: The proposed method for automatic corneal ulcer classification is introduced in Section 2. Section 3 presents the experimental results in detail. In section 4, we conclude this paper and suggest future work.

## Materials and methods

### Overview

**FIGURE 2:**
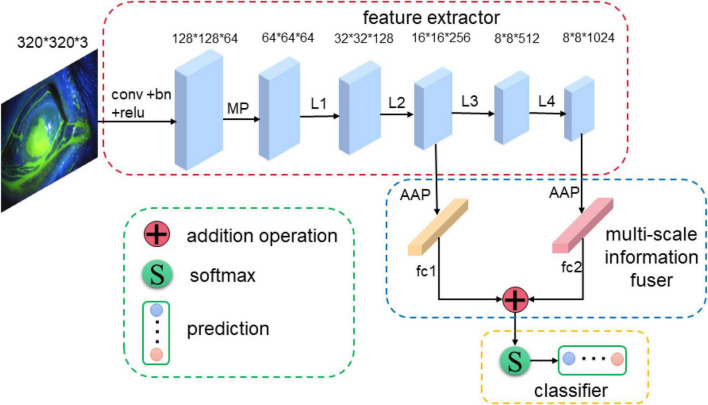
An overview of the proposed MIF-Net for corneal ulcer classification in slit lamp images. The MIF-Net consists of feature extractor, multi-scale information fuser, and classifier, where the feature extractor, multi-scale information fuser, and classifier are in red, blue, and yellow dashed boxes, respectively. “MP,” “GAP,” and “fc” represent max pooling operator, global average pooling operator, fully connected operator, respectively.

The proposed MIF-Net for corneal ulcer classification is shown in Figure 2, which consists of three main parts: feature extractor, multi-scale information fuser, and classifier with label smoothing. Firstly, the feature extractor is used to extract spatial features from the input cornea ulcer image. Then, the multi-scale information fuser is adopted to fuse the classification information from the two different layers of feature extractor. Finally, the label smoothing strategy is applied to the final corneal ulcer classification.

### Feature extractor

To achieve corneal ulcer classification, a backbone network needs to be constructed for feature extraction. We have firstly compared several common backbone networks in image classification, including ResNet (He et al., 2016), DenseNet (Huang et al., 2017), Inception (Ioffe and Szegedy, 2015; Szegedy et al., 2016, 2017), EfficientNet (Tan and Le, 2019), ResNext (Xie et al., 2017), VGGNet (Simonyan and Andrew, 2014). In addition, the above backbone networks have been studied and analyzed in detail. Firstly, experimental results in Tables 2, 3 show the performance of ResNet50 (He et al., 2016), DenseNet121 (Huang et al., 2017), InceptionV3 (Szegedy et al., 2016), ResNext50 (Xie et al., 2017), and VGG16 (Simonyan and Andrew, 2014) is better and comparable. Secondly, compared with ResNet50 (He et al., 2016), InceptionV3 (Szegedy et al., 2016), ResNext50 (Xie et al., 2017), and VGG16 (Simonyan and Andrew, 2014), DenseNet121 (Huang et al., 2017) uses dense connections to encourage features refuse, reduce the number of network parameters greatly, where each front layer function is used as the input of the latter layer. Therefore, considering the balance of model performance and simplicity, we choose DenseNet121 as the backbone network for feature extraction, where the global average pooling layer and the fully connected layer in original version are removed. In addition, many previous studies have shown transfer learning is an effective strategy to speed up the training convergence and improve the classification performance (Pan and Yang, 2010; He et al., 2016; Huang et al., 2017; Tan et al., 2018). Therefore, transfer learning is used to help model training in this study.

**TABLE 2:** The results of comparable experiments on general classification of corneal ulcers.

Methods	W_R (%)	W_P (%)	W_F1 (%)	Kappa (%)	Parameters (M)
ResNet18 (He et al., 2016)	83.28 ± 2.49	83.20 ± 2.25	83.00 ± 2.43	76.72 ± 3.88	11.1781
ResNet34 (He et al., 2016)	83.98 ± 2.75	84.06 ± 2.76	83.80 ± 2.78	76.84 ± 1.20	21.2862
ResNet50 (He et al., 2016)	84.26 ± 2.31	84.32 ± 2.01	84.00 ± 2.21	78.03 ± 3.49	23.5142
DenseNet169 (Huang et al., 2017)	80.33 ± 2.31	70.58 ± 1.84	75.03 ± 1.90	66.15 ± 7.36	12.4895
InceptionResNetV2 (Ioffe and Szegedy, 2015)	83.98 ± 2.51	83.69 ± 2.54	83.56 ± 2.57	74.66 ± 2.17	54.3111
InceptionV3 (Szegedy et al., 2016)	84.68 ± 1.91	84.42 ± 2.09	84.19 ± 1.90	74.84 ± 4.82	21.7917
InceptionV4 (Szegedy et al., 2017)	82.29 ± 2.05	82.32 ± 2.07	82.19 ± 1.99	74.20 ± 1.67	41.1472
ResNxt50 (Xie et al., 2017)	84.82 ± 2.19	84.67 ± 2.16	84.57 ± 2.04	78.63 ± 1.94	22.9861
SE_ResNet50 (Hu et al., 2018)	85.96 ± 2.40	86.83 ± 0.92	86.56 ± 0.97	77.50 ± 1.76	26.0452
SE_ResNext50 (Hu et al., 2018)	83.71 ± 1.08	83.33 ± 1.44	83.03 ± 1.11	74.35 ± 2.11	25.5170
VGG16 (Szegedy et al., 2016)	84.69 ± 1.96	84.34 ± 2.07	84.38 ± 1.95	79.06 ± 3.57	134.2637
EfficientNetB2 (Ioffe and Szegedy, 2015)	81.88 ± 2.24	81.64 ± 2.77	80.87 ± 2.83	72.91 ± 4.91	7.7095
EfficientNetB4 (Ioffe and Szegedy, 2015)	81.75 ± 1.94	81.70 ± 2.47	80.99 ± 2.57	73.19 ± 6.14	17.5594
LmNet (Wan et al., 2021)	85.52 ± 2.24	85.64 ± 2.34	85.17 ± 2.25	78.71 ± 3.25	23.6875
Backbone	84.39 ± 4.05	84.38 ± 4.28	84.25 ± 4.24	78.42 ± 6.31	**6.9569**
Proposed	**87.07 ± 1.98**	**86.93 ± 2.15**	**86.82 ± 2.07**	**81.49 ± 3.25**	6.9577

Bold values indicate the best performance.

**TABLE 3:** The results of comparable experiments on specific classification of corneal ulcers.

Methods	W_R (%)	W_P (%)	W_F1 (%)	Kappa (%)	Parameters (M)
ResNet18 (He et al., 2016)	81.88 ± 1.82	74.82 ± 3.94	77.76 ± 2.76	53.23 ± 9.08	11.1781
ResNet34 (He et al., 2016)	80.19 ± 1.38	72.85 ± 2.95	75.17 ± 3.14	44.15 ± 23.62	21.2862
ResNet50 (He et al., 2016)	81.18 ± 0.51	72.98 ± 1.73	76.49 ± 0.79	54.25 ± 5.25	23.5142
DenseNet169 (Huang et al., 2017)	80.77 ± 1.41	73.24 ± 2.09	75.84 ± 2.39	64.95 ± 10.54	12.4895
InceptionResNetV2 (Ioffe and Szegedy, 2015)	81.88 ± 1.57	76.32 ± 5.42	78.26 ± 3.79	53.06 ± 16.65	54.3111
InceptionV3 (Szegedy et al., 2016)	81.74 ± 1.93	77.52 ± 3.75	79.13 ± 2.59	63.11 ± 8.05	21.7917
InceptionV4 (Szegedy et al., 2017)	81.74 ± 1.11	78.22 ± 3.95	79.30 ± 2.40	63.00 ± 10.40	41.1472
ResNxt50 (Xie et al., 2017)	82.02 ± 2.18	77.66 ± 4.31	78.95 ± 3.72	62.29 ± 9.21	22.9861
SE_ResNet50 (Hu et al., 2018)	81.46 ± 2.58	76.98 ± 4.16	78.71 ± 3.36	57.47 ± 12.53	26.0452
SE_ResNext50 (Hu et al., 2018)	81.87 ± 1.94	78.25 ± 6.68	79.04 ± 4.25	54.99 ± 21.31	25.5170
VGG16 (Szegedy et al., 2016)	81.74 ± 0.56	75.69 ± 4.08	78.17 ± 2.10	57.20 ± 14.30	134.2637
EfficientNetB2 (Ioffe and Szegedy, 2015)	81.17 ± 1.87	73.05 ± 2.45	76.30 ± 2.28	51.04 ± 10.19	7.7095
EfficientNetB4 (Ioffe and Szegedy, 2015)	81.88 ± 1.48	75.18 ± 4.80	78.12 ± 3.34	58.37 ± 11.86	17.5594
LmNet (Wan et al., 2021)	82.42 ± 0.89	74.68 ± 2.30	78.23 ± 1.09	61.60 ± 8.97	23.6875
Backbone	81.45 ± 1.48	74.88 ± 4.10	77.28 ± 2.32	55.39 ± 12.17	**6.9569**
Proposed	**83.84 ± 1.61**	**78.32 ± 2.26**	**80.52 ± 2.25**	**72.06 ± 6.75**	6.9577

Bold values indicate the best performance.

### Multi-scale information fuser

As we know, deep networks have brought performance gains in computer vision tasks, which can extract global feature with deep semantic information. However, many empirical evidences suggest that the performance improvement cannot be achieved by simply stacking more layers (Shen et al., 2016). In addition, most of convolutional neural networks (CNNs) based image classification methods only use the final global feature extracted by the feature extractor for classification, and the shallow local feature does not directly participate in the network training, which may lead to the neglect of some important details for the classification of corneal ulcer. In addition, the current classification, detection and segmentation networks usually use convolutional neural networks to extract the characteristics of objects through layer by layer abstraction. The deeper the network is, the larger the receptive field is, the stronger the semantic information representation ability is, and it is suitable for processing large objects (Chen et al., 2017; George et al., 2018). However, the lower the resolution of the feature map, the weaker the geometric information representation ability is. In the shallow layer of the network, the smaller the down sampling multiple, the smaller the receptive field is, the ability to represent geometric details is strong, and it is suitable for processing small targets (Zhao et al., 2017; George et al., 2018). Although the resolution is high, the ability to represent semantic information is weak. Based the above theory and analysis, the challenge for corneal ulcer classification in this study that different categories of corneal ulcers present lesions of different shapes and sizes can just correspond to the deep and shallow features of the convolution neural network. For example, for point-like corneal ulcers and point-flaky mixed corneal ulcers and type1 corneal ulcers with micro punctate, the feature map is continuously down-sampled through the pooling layer in the CNN, which may lead to the loss of lower resolution features related to the category, so it is very important to retain shallow feature information. For flaky corneal ulcers and type2, type3, type4 corneal ulcers with macro punctate or lager patch, the high-level semantic information is more conducive to improving the classification accuracy due to its relatively large target size. Therefore, inspired by the deep supervised learning and multi-scale feature fusion strategy, which can combine the shallow edge information and deep semantic information to improve the classification performance, we design a new multi-scale information fuser to improve performance with a small increase in the number of model parameters, which is shown in Figure 2. As can be seen from Figure 2, the feature maps of the second and fourth stages of DenseNet121 are fed to two global average pooling layers, respectively, and then the fully connected layers are used to convert them into probability distributions. Finally, we fuse these two predictions to generate the final prediction as shown in Equation 1. Therefore, predictions generated from different levels can supervise the model training.


(1) $$P=f\,\left(P_{1},P_{2}\right)$$

where *P*_*1*_ and *P*_*2*_ are the prediction results of the second and fourth stages of DenseNet121, *P* is the corresponding information fusion results. In addition, *f*(⋅) denotes the information fusion operation.

### Label smoothing strategy

As can be observed from Figure 1 and Table 1, corneal ulcer images have the characteristics of large intra-class diversity and high inter-class similarity to a certain extent. The traditional cross-entropy loss function only calculates the loss that the predicted value is the real class, which may lead to poor classification performance of the model in this study due to inter-class similarity and intra-class difference. Therefore, we introduce label smoothing strategy into the cross-entropy loss function to reduce the similarity between classes and difference within classes and improve the generalization of the model, which can alleviate the overconfidence problem caused by the traditional cross-entropy loss function.

Suppose D is a classification dataset with M samples (*x*_*i*_,*y*_*i*_) (*i* = 1, 2, …, M), where *x_i_* and *y_i_* represent an input image and its corresponding category label, respectively. A standard multi-classification problem is to predict the probability of the input image *x_i_*, belonging to category k (*y*_*i*_ = *k*). The category k is encoded by one-hot labels as a vector *t* = (0, 0, …, 0, 1, 0, …, 0), where only *t_k_* is 1 and all others are 0. This characteristic encourages the model to learn in the direction with the greatest difference between the correct label and the wrong label, which means only the loss of the correct label position is calculated in the optimization process of the model. However, when the training data is small, and the inter-class similarity and intra-class differences is relatively large, it may cause the network to be overfitting (Wan et al., 2021). To solve the above problems and inspired by previous study (Szegedy et al., 2017; Müller et al., 2019), label smoothing is introduced in this study. It is a regularization strategy, which mainly adds noise through soft one-hot to reduce the weight of the true label category and slightly increases the penalty for the wrong label category in the training of the model, and ultimately reduces the risk of overfitting. The label smoothing strategy used in this study is as follows:


(2) $$t^{{}^{\prime }}=t*\left(1-\varepsilon \right)+I*\frac{\varepsilon }{N}$$

where *t* and *t*′ represent the one-hot labels before and after label smoothing. ε is a random number between 0.1 and 0.2, which can be regarded as noise introduced in the fixed distribution. *I* is a matrix with the same dimension as *t*, and its element values are all one. N is the total number of categories.

### Loss function

In this study, we propose a multi-scale information fusion network (MIF-Net), which takes the original cornea ulcer images as input. In addition, considering the similarity between classes and differences within classes, label smoothing strategy is used in network optimization. Based on the analysis, the cross-entropy loss function based on label smoothing strategy is used and defined as follow:


(3) $$\text{L}=-\frac{1}{m}\sum_{i=1}^{m}\sum_{k=1}^{K}f\left(t_{i}=k\right)*\log\left(p\left(k|x_{i}\right)\right)+\left(1-f\left(t_{i}=k\right)\right)*\text{log}\left(1-p\left(k|x_{i}\right)\right)$$

where m is the number of samples in per mini-batch, *t_i_* is the class label of the input image *x_i_*. *f*(⋅) is an indicator function, which is one if *t_i_* equals k (*k* = 1, 2, …, K).

## Experiments and results

In this section, we first introduce the experimental dataset in detail. Then, the experimental setup will be described, including imaging processing, the parameter settings in the training phase and evaluation metrics in the testing phase. Finally, we will give the detailed experimental results and the corresponding analysis.

### Dataset

In this study, the SUSTech-SYSU public dataset (Deng et al., 2020) is used to evaluate the proposed MIF-Net, which contains a total of 712 fluorescein staining images with ground truth annotated in image-wise by three experienced ophthalmologists from Zhongshan Ophthalmic Center at Sun Yat-sen University. The fluorescein staining image with a resolution of 2592 × 1728 pixels contains only one cornea, which is completely presented in the image, roughly in the center of the visual field. The labeling of corneal ulcers classification is based on the symptoms described in Table 1. It can be seen from Table 1 that the category distribution is unbalanced, where most cornea ulcer data are point-like corneal ulcers, point-flaky mixed corneal ulcers, and type4 corneal ulcers and the data of several other corneal ulcers is relatively few. To evaluate the effectiveness of the proposed MIF-Net, a 5-fold cross-validation strategy is adopted, where the whole dataset is randomly divided into 5 subsets of the almost same size according to the proportion of the number of each category.

### Experimental setup

#### Image processing

To reduce the computational cost and improve the computational efficiency of the model, all the images are resized to 320 × 320 by bilinear interpolation and normalized to Lopes et al. (2019). In addition, online data augmentation, including random rotation 30°, horizontal flipping, and vertical flipping, is adopted to prevent over-fitting and improve the robust ability of the model.

#### Parameter setting

The proposed MIF-Net is performed on the public platform Pytorch. We use A NVIDIA GTX Titan X GPU with 12GB memory to train the model with back-propagation algorithm by minimizing the loss function as illustrated in Equation 3. The Adam was used as the optimizer, where both initial learning rate and weight decay are set to 0.0001. The batch size and epoch are set to 16 and 50, respectively. To ensure fairness, all the networks in this study are trained with same optimization schemes and we save the best model on validation set. The code of the proposed MIF-Net will be released in: https://github.com/linquanlv0915/MIF-Net.

#### Evaluation metrics

Considering the category imbalance of the dataset shown in Table 1 and to comprehensively and fairly evaluate the classification performance of different methods, four common evaluation indicators are used (Peng et al., 2021, 2022b), including weighted-average recall (W_R), weighted-average precision (W_P), weighted-average F1 score (W_F1), Kappa index (McHugh, 2012).

### Results

In this study, we propose a classification network named MIF-Net with label smoothing for two classification patterns of corneal ulcer, including general ulcer pattern and specific ulcer pattern. In our experiments, we evaluate performance of the proposed method on the 712 fluorescein staining images by using 5-fold cross-validation strategy, which randomly divided the whole dataset into 5 subsets according to the proportion of the number of each category. In each experiment, model was trained with 4 subsets and test on the remaining one subset. The experiments were repeated 5 times with each of the 5 subsets used exactly once as the testing set and the other four subsets as training set and the final experimental results were averaged over all the experiments. Next, a series of comparison experiments and ablation experiments are presented and analyzed in detailed. For convenience, the basic DenseNet121 pretrained on ImageNet is called Backbone.

#### Results on general ulcer pattern

As can be seen from Table 1, the corneal ulcers can be divided into three categories according to the general ulcer pattern, namely point-like corneal ulcers, point-flaky mixed corneal ulcers and flaky corneal ulcers. Table 2 shows the quantitative results of different methods. We compare the proposed method with other excellent CNN based classification networks, including ResNet18 (He et al., 2016), ResNet34 (He et al., 2016), ResNet50 (He et al., 2016), DenseNet169 (Huang et al., 2017), InceptionResNetV2 (Ioffe and Szegedy, 2015), InceptionV3 (Szegedy et al., 2016), InceptionV4 (Szegedy et al., 2017), ResNext50 (Xie et al., 2017), SE_ResNet50 (Hu et al., 2018), SE_ResNext50 (Hu et al., 2018), VGG16 (Simonyan and Andrew, 2014), EfficientNetB2 (Tan and Le, 2019), and EfficientNetB4 (Tan and Le, 2019). It can be seen from Table 2 that our method achieves superior performance in term of all evaluation metrics.

First, compared with Backbone, the performance of the proposed method has been greatly improved, which improves the W_R, W_P, W_F1, and Kappa by 3.92, 3.02, 3.05, and 3.91%, respectively, and achieves 87.07% for W_R, 86.93% for W_P, 86.82% for W_F1, and 81.49% for Kappa. Then, compared with other state-of-the-art classification networks, our MIF-Net with label smoothing gets an overall improvement in terms of all indicators with comparable or less model complexity. For example, compared with the best performance among the comparison classification networks (SE_ResNet50), the proposed method with less model parameters improves the W_R, W_P, W_F1, and Kappa by 1.29, 0.12, 0.30, and 5.15%, respectively. In addition, compared with EfficientNetB2 with comparable model parameters, our proposed method has also made great improvement in terms of all evaluation metrics. It is worth noting that the proposed method is also compared with a recent study on multi-scale feature fusion and label smoothing, which is proposed for remote sensing classification and is named LmNet (Wan et al., 2021). LmNet takes pre-trained ResNext50 as backbone and combines channel attention, multi-scale feature fusion and label smoothing. As can be seen from Table 2, the proposed MIF-Net with label smoothing outperforms LmNet on all metrics, which improves the W_R by 1.75%. Moreover, the model complexity of our method is less than that of LmNet. These results demonstrate the effectiveness of the proposed method in the general classification pattern of corneal ulcers.

#### Results on specific ulcer pattern

It can be seen from Table 1, the corneal ulcers can be divided into five categories according to the specific ulcer pattern, namely type0, type1, type2, type3, and type4, respectively. Similar to the general ulcer pattern, a series of comparison experiments with the other state-of-the-art classification networks are conducted and the quantitative results are illustrated in Table 3. As can be observed from Table 3, the performance of ResNet34 is the worst, while the performance of ResNext50 is the second best. In addition, compared to the Backbone, our proposed method gets an overall improvement in term of all evaluation indicators and improves the W_R, W_P, W_F1, and Kappa by 2.93, 4.59, 4.19, and 30.10%, respectively. It can be seen from Table 3 that the performance of the proposed method is better than other CNNs based classification networks, which achieves 83.84% for W_R, 78.32% for W_P, 80.52% for W_F1, and 72.06% for Kappa, respectively. Similarly, we also compare the proposed method with LmNet (Wan et al., 2021). As can be observed from Table 3, compared to LmNet, our proposed method achieves better performance, which improves the W_R, W_P, W_F1, and Kappa by 2.93, 3.64, 2.93, and 16.98%, respectively. The experimental results prove the effectiveness of the proposed method for the specific classification pattern of corneal ulcers in slit lamp images.

### Ablation experiments

#### Ablation study for multi-scale information fuser

As can be seen from Figure 2, we propose a simple and effective multi-scale information strategy in this study. In this section, we explore the influence of different information fusion strategies on the corneal ulcer classification and conduct the ablation experiments as shown in Table 4, where “Backbone + Fusion_add_234” denotes we fuse the predictions of second, third and fourth stages of DenseNet121 by addition operation to generate the final prediction and the meaning of the others is similar. In particular, “Backbone + Fusion_add_24” is our proposed fusion strategy in this study. Taking the general classification of corneal ulcers for example, it can be seen from Table 4 that compared to the Backbone, the model’s performance with all multi-scale information fusion strategies can get an overall improvement in terms of all metrics, which proves multi-scale information fusion can improve the model’s classification performance in this study. It is worth noting that the information fusion based on addition operation performs better than information fusion based on maximum operation and concatenation operation as show in Table 4. Firstly, for the corneal ulcer classification of general pattern, compared to Backbone, the performance of “Backbone + Fusion_add_24” improves by 2.33, 2.76, 2.41, and 3.03% for W_R, W_P, W_F1, and Kappa, respectively. In addition, for the corneal ulcer classification of specific pattern, the introduction of multi-scale information fusion based on addition also achieves better classification performance. As shown in Table 5, compared with the Backbone, the W_R, W_P, W_F1, and Kappa increase from 81.45, 74.88, 77.28, and 55.39% to 82.44, 75.96, 79.11, and 60.54%, respectively, which benefits from the fact that multi-scale infusion fusion can fully integrate shallow detailed information and deep semantic information to improve the multi-scale feature representation ability of the model. These results indicate the effectiveness of the proposed multi-scale information fuser.

**TABLE 4:** The results of ablation experiments on general classification of corneal ulcers.

Methods	W_R (%)	W_P (%)	W_F1 (%)	Kappa (%)	Parameters (M)
Backbone	84.39 ± 4.05	84.38 ± 4.28	84.25 ± 4.24	78.42 ± 6.31	**6.9569**
Backbone + Fusion_add_234	85.52 ± 2.80	85.37 ± 3.01	85.24 ± 2.87	78.69 ± 4.91	6.9592
Backbone + Fusion_add_34	85.94 ± 2.27	85.7 ± 2.02	85.50 ± 2.01	80.26 ± 1.77	6.9585
Backbone + Fusion_add_24	**86.36 ± 2.70**	**86.71 ± 2.92**	**86.28 ± 2.67**	**80.80 ± 3.11**	6.9577
Backbone + Fusion_max_234	85.25 ± 2.22	85.18 ± 2.43	84.82 ± 2.18	79.28 ± 4.34	6.9592
Backbone + Fusion_max_34	85.24 ± 2.96	85.04 ± 2.86	85.07 ± 2.93	79.92 ± 3.31	6.9585
Backbone + Fusion_max_24	84.69 ± 2.99	84.57 ± 2.80	84.49 ± 2.95	78.74 ± 6.67	6.9577
Backbone + Fusion_Concatenation_234	85.23 ± 3.61	85.15 ± 3.32	85.02 ± 3.37	80.29 ± 3.16	6.9577
Backbone + Fusion_Concatenation _34	84.96 ± 3.58	84.94 ± 3.21	84.64 ± 3.51	79.67 ± 5.97	6.9585
Backbone + Fusion_Concatenation _24	85.38 ± 3.79	84.85 ± 4.07	84.63 ± 4.17	78.58 ± 8.71	6.9592
Backbone + LS	86.21 ± 3.55	86.15 ± 3.40	86.07 ± 3.50	80.15 ± 6.49	6.9577

Bold values indicate the best performance.

**TABLE 5:** The results of ablation experiments on specific classification of corneal ulcers.

Methods	W_R (%)	W_P (%)	W_F1 (%)	Kappa (%)	Parameters (M)
Backbone	81.45 ± 1.48	74.88 ± 4.10	77.28 ± 2.32	55.39 ± 12.17	**6.9569**
Backbone + Fusion_add_24	82.44 ± 1.23	75.96 ± 2.10	79.11 ± 1.22	60.54 ± 8.74	6.9577
Backbone + LS	**83.00 ± 1.29**	**77.68 ± 2.34**	**79.88 ± 1.63**	**65.85 ± 6.24**	6.9577

Bold values indicate the best performance.

#### Ablation study for label smoothing strategy

It can be observed from Tables 4, 5, the introduction of label smoothing strategy (Backbone + LS) also gets an overall improvement in terms of all evaluation indexes for two corneal ulcer classification tasks. Taking the corneal ulcer classification of general pattern for example, compared with the Backbone, the W_R, W_P, W_F1, and Kappa of the Backbone+LS increase by 2.16, 2.10, 2.16, and 2.21%, respectively, which may benefit the fact that the introduction of label smoothing can reduce the impact of similarity between classes and intra-class difference on classification performance. The results prove the effectiveness of label smoothing strategy in this study.

## Conclusion and discussion

Accurate corneal ulcer classification is still a challenging task due to its complex pathology, noise interference and the similar pathological morphology and distribution of different types of corneal ulcer. In this study, to tackle these problems, a novel classification network named MIF-Net with label smoothing is proposed for corneal ulcer classification. Firstly, to avoid the loss of lower resolution features related to category caused by down sampling, a multi-scale information fuser is designed to fully integrate shallow local information and depth global features to improve the multi-scale information representation ability of the model. Then, to reduce the influence of inter-class similarity and intra-class diversity on feature representation, another learning strategy named label smoothing is developed to improve the generalization of the model, which is a modification of the loss function. The ablation experiments show that both multi-scale information fuser and label smoothing strategy can improve the classification performance. Compared with other state-of-the-art CNN-based classification networks, the classification performance of the proposed MIF-Net with label smoothing has been improved, as shown in Tables 2, 3.

**FIGURE 3:**
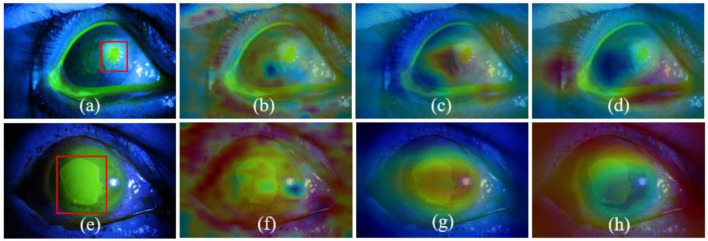
Visualization results of CAM. Panels **(a,e)** are the original images, where the ulcer-related pathologies are in red boxes. Panels **(b,c,f,g)** are the heat maps of the second and fourth stages of the proposed MIF-Net, while the panels **(d,h)** is the heat maps of the fourth stage of Backbone.

To further illustrate the effectiveness of the proposed MIF-Net and increase the interpretability of CNN, we apply the “class activation mapping” technology (Zhou et al., 2016) to obtain the heat maps of fluorescein staining images with different corneal ulcer categories for the qualitative analysis, which calculates the convolutional outputs of the second and fourth stages and visualizes the class-discriminative regions concerned by the network. As can be seen from Figures 3a,b,e,f, the features extracted by the proposed method in the shallow layer are local detailed information, while the features extracted from the last layer are global semantic information as shown in Figures 3c,g. In addition, it can be observed from Figures 3a,c–e,g,h that compared to Backbone, our method can focus on the location of key information related to different corneal ulcer categories, which may benefit from multi-scale information fusion and label smoothing strategy. These results demonstrate that compared with Backbone, the proposed method can adaptively focus on target-related area of corneal ulcer images and efficiently improve the classification performance of corneal ulcer.

In conclusion, the proposed MIF-Net with label smoothing holds promise for corneal ulcer classification in slit lamp images. We believe that our proposed method can also be applied to other medical image classification tasks, which requires further exploration and verification. However, there is still a limitation in this study that all comparisons between the proposed method and other classification networks are based on the limited data from SUSTech-SYSU dataset. We believe that if more data are available, the performance of our method will be further improved. Therefore, in the near future, we will collect more fluorescein staining images of corneal ulcers to further evaluate the performance of the proposed method and develop relevant semi-supervised algorithms based on MIF-Net to reduce the dependence on labeled data.

## Data availability statement

The datasets presented in this study can be found in online repositories. The names of the repository/repositories and accession number(s) can be found below: https://github.com/CRazorback/The-SUSTech-SYSU-dataset-for-automatically-segmenting-and-classifying-corneal-ulcers.

## Ethics statement

The studies involving human participants were reviewed and approved by the Zhongshan Ophthalmic Centre Ethics Committee of Sun Yat-sen University. The patients/participants provided their written informed consent to participate in this study.

## Author contributions

LL designed the study, conducted most of experiments, analyzed the experimental results, and drafted the manuscript. MP conducted some of experiments, reviewed and revised the manuscript. XW and YW reviewed the manuscript and conducted some of experiments. All authors contributed to the article and approved the submitted version.

## References

[B1] Akram A. Debnath R. (2019). “An efficient automated corneal ulcer detection method using convolutional neural network,” in *Proceedings of the 2019 22nd international conference on computer and information technology* (Dhaka: IEEE), 1–6. 10.1109/ICCIT48885.2019.9038389

[B2] Chen L. Papandreou G. Schroff F. Adam H. (2017). Rethinking atrous convolution for semantic image segmentation. *arXiv* [Preprint]. arXiv:1706.05587.

[B3] Chun Y. S. Yoon W. B. Kim K. G. Park I. K. (2014). Objective assessment of corneal staining using digital image analysis. *Invest. Ophthalmol. Vis. Sci.* 55 7896–7903. 10.1167/iovs.14-15618 25406292

[B4] Cohen E. J. Laibson P. R. Arentsen J. J. Clemons C. S. (1987). Corneal ulcers associated with cosmetic extended wear soft contact lenses. *Ophthalmology* 94 109–114. 10.1016/S0161-6420(87)33491-8 3472135

[B5] Deng L. Huang H. Yuan J. Tang X. (2018b). Automatic segmentation of corneal ulcer area based on ocular staining images. Medical imaging 2018: Biomedical applications in molecular, structural, and functional imaging. *Int. Soc. Opt. Photonics* 10578:105781D. 10.1117/12.2293270

[B6] Deng L. Huang H. Yuan J. Tang X. (2018a). “Superpixel based automatic segmentation of corneal ulcers from ocular staining images,” in *Proceedings of the IEEE 23rd international conference on digital signal processing* (Shanghai: IEEE), 1–5. 10.1109/ICDSP.2018.8631795

[B7] Deng L. Lyu J. Huang H. Deng Y. Yuan J. Tang X. (2020). The SUSTech-SYSU dataset for automatically segmenting and classifying corneal ulcers. *Sci. Data* 7:23. 10.1038/s41597-020-0360-7 31959768 PMC6971241

[B8] Deswal J. Arya S. K. Raj A. Bhatti A. (2017). A case of bilateral corneal perforation in a patient with severe dry eye. *J. Clin. Diagn. Res.* 11 ND01–ND02. 10.7860/JCDR/2017/24149.9645 28571178 PMC5449824

[B9] Diamond J. Leeming J. Coombs G. Pearman J. Sharma A. Illingworth C. (1999). Corneal biopsy with tissue micro homogenisation for isolation of organisms in bacterial keratitis. *Eye* 13 545–549. 10.1038/eye.1999.135 10692928

[B10] George L. C. Schroff P. F. Adam H. (2018). “Encoder-decoder with atrous separable convolution for semantic image segmentation,” in *Proceedings of the conference on computer vision and pattern recognition* (Salt Lake City, UT: IEEE), 1–11.

[B11] He K. Zhang X. Ren S. Sun J. (2016). “Deep residual learning for image recognition,” in *Proceedings of the IEEE conference on computer vision and pattern recognition* (Las Vegas, NV: IEEE), 770–778. 10.1109/CVPR.2016.90

[B12] Hu J. Shen L. Sun G. (2018). “Squeeze-and-excitation networks,” in *Proceedings of the IEEE conference on computer vision and pattern recognition* (Salt Lake City, UT: IEEE), 7132–7141. 10.1109/CVPR.2018.00745

[B13] Huang G. Liu Z. Laurens V. D. W. Weinberger K. Q. (2017). “Densely connected convolutional networks,” in *Proceedings of the IEEE conference on computer vision and pattern recognition* (Honolulu, HI: IEEE), 4700–4708. 10.1109/CVPR.2017.243

[B14] Ioffe S. Szegedy C. (2015). “Batch normalization: Accelerating deep network training by reducing internal covariate shift,” in *Proceedings of the international conference on machine learning*, Mountain View, CA.

[B15] Kaufman H. (1960). The diagnosis of corneal herpes simplex infection by fluorescent antibody staining. *Arch. Ophthalmol.* 64 382–384. 10.1001/archopht.1960.01840010384009 14404856

[B16] Khanal S. Tomlinson A. McFadyen A. Diaper C. Ramaesh K. (2008). Dry eye diagnosis. *Invest. Ophthalmol. Vis. Sci.* 49 1407–1414. 10.1167/iovs.07-0635 18385057

[B17] Kumar A. Thirumalesh M. (2013). Use of dyes in ophthalmology. *J. Clin. Ophthalmol. Res.* 1 55–58. 10.4103/2320-3897.106288

[B18] Lee C. Xie S. Gallagher P. Zhang Z. Tu Z. (2015). Deeply-supervised nets. *Artif. Intell. Stat.* 39 562–570. 31035673

[B19] Liu H. Wong D. W. K. Fu H. Xuet Y. Liu J. (2018). “DeepAMD: Detect early age-related macular degeneration by applying deep learning in a multiple instance learning framework,” in *Proceedings of the Asian conference on computer vision* (Cham: Springer), 625–640. 10.1007/978-3-030-20873-8_40

[B20] Lopes B. T. Eliasy A. Ambrosio R. (2019). Artificial intelligence in corneal diagnosis: Where are we? *Curr. Ophthalmol. Rep.* 7 204–211. 10.1007/s40135-019-00218-9

[B21] Ma C. Mu X. Sha D. (2019). Multi-layers feature fusion of convolutional neural network for scene classification of remote sensing. *IEEE Access* 7 121685–121694. 10.1109/ACCESS.2019.2936215

[B22] Manikandan P. Abdel-hadi A. Randhir Babu Singh Y. Revathi R. Anita R. Banawas S. (2019). Fungal keratitis: Epidemiology, rapid detection, and antifungal susceptibilities of *Fusarium* and *Aspergillus* isolates from corneal scrapings. *Biomed Res. Int.* 2019:6395840. 10.1155/2019/6395840 30800674 PMC6360544

[B23] McHugh M. L. (2012). Interrater reliability: The kappa statistic. *Biochem. Med.* 22 276–282. 10.11613/BM.2012.031 PMC3900052 23092060

[B24] Morgan P. B. Carole M. C. (2009). Corneal staining: Do we really understand what we are seeing. *Cont. Lens Anterior Eye* 32 48–54. 10.1016/j.clae.2008.09.004 19181563

[B25] Müller R. Kornblith S. Hinton G. E. (2019). When does label smoothing help?. *arXiv* [Preprint]. arXiv:1906.02629.

[B26] Pan S. J. Yang Q. (2010). A survey on transfer learning. *IEEE Trans. Knowl. Data Eng.* 22 1345–1359. 10.1109/TKDE.2009.191

[B27] Peng Y. Chen Z. Zhu W. Shi F. Wang M. Zhou Y. (2022a). ADS-net: Attention-awareness and deep supervision based network for automatic detection of retinopathy of prematurity. *Biomed. Opt. Express* 13 4087–4101. 10.1364/BOE.461411 36032570 PMC9408258

[B28] Peng Y. Chen Z. Zhu W. Shi F. Wang M. Zhou Y. (2022b). Automatic zoning for retinopathy of prematurity with semi-supervised feature calibration adversarial learning. *Biomed. Opt. Express* 13 1968–1984. 10.1364/BOE.447224 35519283 PMC9045915

[B29] Peng Y. Zhu W. Chen Z. Shi F. Wang M. Zhou Y. (2022c). AFENet: Attention fusion enhancement network for optical disc segmentation of prematurity infants. *Front. Neurosci.* 13:836327. 10.3389/fnins.2022.836327 35516802 PMC9063315

[B30] Peng Y. Zhu W. Chen F. Xiang D. Chen X. (2020). Automated retinopathy of prematurity screening using deep neural network with attention mechanism. *Proc. Med. Imaging* 11313 1131321–1131327. 10.1117/12.2548290 30166272

[B31] Peng Y. Zhu W. Chen Z. Wang M. Geng L. Yu K. (2021). Automatic staging for retinopathy of prematurity with deep feature fusion and ordinal classification strategy. *IEEE Trans. Med. Imaging* 40 1750–1762. 10.1109/TMI.2021.3065753 33710954

[B32] Peterson R. Wolffsohn J. (2009). Objective grading of the anterior eye. *Optom. Vis. Sci.* 86 273–278. 10.1097/OPX.0b013e3181981976 19165123

[B33] Pritchard N. Young G. Coleman S. Hunt C. (2003). Subjective and objective measures of corneal staining related to multipurpose care systems. *Cont. Lens Anterior Eye* 26 3–9. 10.1016/S1367-0484(02)00083-8 16303491

[B34] Schweitzer N. (1967). A fluorescein colored polygonal pattern in the human cornea. *Arch. Ophthalmol.* 77 548–553. 10.1001/archopht.1967.00980020550021 6022725

[B35] Shen L. Lin Z. Huang Q. (2016). “Relay backpropagation for effective learning of deep convolutional neural networks,” in *Proceedings of the European conference on computer vision* (Cham: Springer), 467–482. 10.1007/978-3-319-46478-7_29

[B36] Simonyan K. Andrew Z. (2014). Very deep convolutional networks for large-scale image recognition. arXiv [Preprint]. arXiv:1409.1556.

[B37] Smith T. (2004). *BMA AZ family medical encyclopedia.* London: Dorling Kindersley Ltd.

[B38] Sun Q. Deng L. Liu J. Huang H. Yuan J. Tang X. (2017). “Patch-based deep convolutional neural network for corneal ulcer area segmentation,” in *Fetal, infant and ophthalmic medical image analysis*, eds Jorge Cardoso M. Arbel T. Melbourne A. Bogunovic H. Moeskops P. Chen X. (Cham: Springer), 101–108. 10.1007/978-3-319-67561-9_11

[B39] Szegedy C. Ioffe S. Vanhoucke V. Alemi A. (2017). “Inception-v4, Inception-resnet and the impact of residual connections on learning,” in *Proceedings of the thirty-first AAAI conference on artificial intelligence*, California, CA. 10.1609/aaai.v31i1.11231

[B40] Szegedy C. Vanhoucke V. Ioffe S. Shlens J. Wojna Z. (2016). “Rethinking the inception architecture for computer vision,” in *Proceedings of the IEEE conference on computer vision and pattern recognition* (Las Vegas, NV: IEEE), 2818–2826. 10.1109/CVPR.2016.308

[B41] Tan C. Sun F. Kong T. Zhang W. Yang C. Liu C. (2018). “A survey on deep transfer learning,” in *Proceedings of the international conference on artificial neural networks* (Berlin: Springer), 270–279. 10.1007/978-3-030-01424-7_27

[B42] Tan M. Le Q. V. (2019). Efficientnet: Rethinking model scaling for convolutional neural networks. *arXiv* [Preprint]. arXiv:1905.11946.

[B43] Wan H. Chen J. Huang Z. Feng Y. Zhou Z. Liu X. (2021). Lightweight channel attention and multiscale feature fusion discrimination for remote sensing scene classification. *IEEE Access* 9 94586–94600. 10.1109/ACCESS.2021.3093308

[B44] Wang L. Chen Y. Tu Z. Svetlana L. (2015). Training deeper convolutional networks with deep supervision. *arXiv* [Preprint]. arXiv:1505.02496.

[B45] Wang S. Wang X. Hu Y. Shen Y. Yang Z. Gan M. (2020). Diabetic retinopathy diagnosis using multichannel generative adversarial network with semi-supervision. *IEEE Trans. Autom. Sci. Eng.* 13 1–12.

[B46] Wolffsohn J. Purslow C. (2003). Clinical monitoring of ocular physiology using digital image analysis. *Cont. Lens Anterior Eye* 26 27–35. 10.1016/S1367-0484(02)00062-0 16303494

[B47] Xie S. Girshick R. Dollár P. Tu Z. He K. (2017). “Aggregated residual transformations for deep neural networks,” in *Proceedings of the IEEE conference on computer vision and pattern recognition* (Honolulu, HI: IEEE), 1492–1500. 10.1109/CVPR.2017.634

[B48] Zhang Y. Chen P. Di G. Qi X. Gao H. (2018). Netrin-1 promotes diabetic corneal wound healing through molecular mechanisms mediated via the adenosine 2b receptor. *Sci. Rep.* 8:5994. 10.1038/s41598-018-24506-9 29662125 PMC5902612

[B49] Zhao H. Shi J. Qi X. Wang X. Jia J. (2017). “Pyramid scene parsing network,” in *Proceedings of the IEEE conference on computer vision and pattern recognition* (Honolulu, HI: IEEE), 2881–2890. 10.1109/CVPR.2017.660

[B50] Zhou B. Khosla A. Lapedriza A. Oliva A. Torralba A. (2016). “Learning deep features for discriminative localization,” in *Proceedings of the IEEE conference on computer vision and pattern recognition* (Las Vegas, NV: IEEE), 2921–2929. 10.1109/CVPR.2016.319

